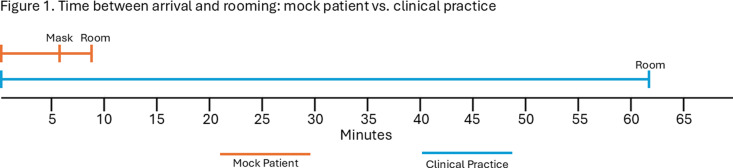# 372 Clean Slate: Enhancing MRSA Decolonization Protocols

**DOI:** 10.1017/ash.2026.10708

**Published:** 2026-06-23

**Authors:** Jacob McAlinn, Marlee Barton, Angela Montgomery, Rohana Bruker, Guillermo Rodriguez Nava, Heather Young, Adam Beitscher

**Affiliations:** 1 Denver Health and Hospital Authority; 2 Denver Health; 3 Denver Health Hospital Authority; 4 Denver Health Medical Center

## Abstract

**Background:** During a measles outbreak in the United States in 2025, health care settings were on high alert for patients who may have been exposed to or infected with this disease. When an infection is found, contact tracing investigations are extensive and require significant labor. Identify, Isolate, Inform (III) has become a standard tool for special pathogen preparedness. Our facility implemented an electronic medical record (EMR) alert for patients reporting fever and rash. The objective of this work is to compare and contrast our experience in drill vs clinical practice. **Methods:** Setting. Denver Health is an acute-care safety net hospital with both adult and pediatric emergency departments, sharing a common security checkpoint for entrance, and a check in and arrival space before patients are triaged into individual waiting rooms. Drill. In March 2025, Denver Health conducted a drill to test the III process in the pediatric emergency department and urgent care center (PEDUC) where care was provided for a mock patient with fever, rash, and cough. Time to masking and time to isolation were collected by a drill observer. Clinical practice. In April 2025, an infant with recent international travel presented to the PEDUC with cough, fever, and rash and was subsequently diagnosed with measles by PCR. Time to isolation was collected via time stamps in the EMR. **Results:** During the drill, fever and rash were documented, and the alert fired appropriately. The mock patient was masked within six minutes of entry and roomed in a negative pressure room within nine minutes of entry. During clinical practice, rash was documented, but the patient was afebrile at triage, and the EMR alert did not fire. The patient spent 62 minutes in the waiting room before being roomed. Contact tracing involved individuals in both the adult and pediatric emergency departments due to the shared airspaces. In total, 149 patients were identified as contacts and were notified of the exposure by public health. No transmission was identified. **Conclusions:** Despite a recent successful drill in the PEDUC, when a patient presented with measles, there were challenges with identification and isolation, as well as structural limitations within the facility and gaps with technology aiding the early identification of patients at risk. Having a real-life situation resulted in an extremely large contact investigation and juxtaposed the recent successful drill, highlighting the need to continue performing “no notice drills” and work on improving each facet of preparedness.

**Figure:**